# Zingerone Suppresses Tumor Development through Decreasing Cyclin D1 Expression and Inducing Mitotic Arrest

**DOI:** 10.3390/ijms19092832

**Published:** 2018-09-19

**Authors:** Jae-Sun Choi, Jaewook Ryu, Woom-Yee Bae, Aron Park, Seungyoon Nam, Ja-Eun Kim, Joo-Won Jeong

**Affiliations:** 1 Department of Anatomy and Neurobiology, College of Medicine, Kyung Hee University, Seoul 02447, Korea; ChoiJS@khu.ac.kr; 2 Department of Biomedical Science, Graduate School, Kyung Hee University, Seoul 02447, Korea; busterray@naver.com (J.R.); woom8875@khu.ac.kr (W.-Y.B.); 3 Department of Health Sciences and Technology, Gachon Advanced Institute for Health Sciences and Technology, Gachon University, Incheon 21936, Korea; parkar13@gmail.com; 4 Department of Genome Medicine and Science, College of Medicine, Gachon University, Incheon 21936, Korea; nams@gachon.ac.kr; 5 Department of Life Sciences, Gachon University, Seongnam 13120, Korea; 6 Gachon Institute of Genome Medicine and Science, Gachon University Gil Medical Center, Incheon 21936, Korea; 7 Department of Pharmacology, College of Medicine, Kyung Hee Univeristy, Seoul 02447, Korea

**Keywords:** zingerone, mitosis arrest, cyclin D1, apoptosis, tumor progression

## Abstract

Cancer cells undergo uncontrolled proliferation resulting from aberrant activity of various cell-cycle proteins. Therefore, despite recent advances in intensive chemotherapy, it is difficult to cure cancer completely. Recently, cell-cycle regulators became attractive targets in cancer therapy. Zingerone, a phenolic compound isolated from ginger, is a nontoxic and inexpensive compound with varied pharmacological activities. In this study, the therapeutic effect of zingerone as an anti-mitotic agent in human neuroblastoma cells was investigated. Following treatment of BE(2)-M17 cells with zingerone, we performed a 3-(4,5-dimethylthiazol-2-yl)-2,5- diphenyltetrazolium bromide (MTT) assay and colony-formation assay to evaluate cellular proliferation, in addition to immunofluorescence cytochemistry and flow cytometry to examine the mitotic cells. The association of gene expression with tumor stage and survival was analyzed. Furthermore, to examine the anti-cancer effect of zingerone, we applied a BALB/c mouse-tumor model using a BALB/c-derived adenocarcinoma cell line. In human neuroblastoma cells, zingerone inhibited cellular viability and survival. Moreover, the number of mitotic cells, particularly those in prometaphase, increased in zingerone-treated neuroblastoma cells. Regarding specific molecular mechanisms, zingerone decreased cyclin D1 expression and induced the cleavage of caspase-3 and poly (ADP-ribose) polymerase 1 (PARP-1). The decrease in cyclin D1 and increase in histone H3 phosphorylated (p)-Ser10 were confirmed by immunohistochemistry in tumor tissues administered with zingerone. These results suggest that zingerone induces mitotic arrest followed by inhibition of growth of neuroblastoma cells. Collectively, zingerone may be a potential therapeutic drug for human cancers, including neuroblastoma.

## 1. Introduction

Cancer is a major cause of death across the world. Cancer cells grow in an uncontrollable way and often invade and spread to other parts of the body. Cancer is characterized by uncontrolled cell proliferation resulting from aberrant cell-cycle activity [1]. The mammalian cell cycle is highly organized and tightly controlled. Proliferation depends on progression through gap 0/1 (G0/G1), synthesis (S), gap 2 (G2), and mitosis (M) phases, regulated by various regulator proteins such as cyclin-dependent kinases (CDKs) [2,3,4]. Because aberrant activation of cell-cycle activators is frequently found in human cancers, cell-cycle regulators are considered attractive targets for cancer treatment [1].

Ginger (*Zingiber officinale*) is the most common spice and fresh herb used throughout the world. Since ancient times, ginger is used in China, Greece, and India for the treatment of colds, headaches, nausea, upset stomach, and diarrhea [5,6,7]. Ginger contains active phenolic compounds such as gingerols, paradols, shogaols, and zingerone. Earlier studies showed that these compounds possess anti-oxidant, anti-angiogenic, anti-atherosclerotic, and anti-cancer properties [7,8,9,10]. Zingerone is primarily present in dry ginger, as gingerol is converted to zingerone during cooking or drying [11]. Zingerone has a methoxy group attached to a benzene ring [12]. Zingerone showed anti-cancer effects in rat colon cancer [13] and anti-angiogenic activity during tumorigenesis [14]. However, although phenolic compounds are suggested to exert chemopreventive effects [7], the anti-cancer effects of zingerone are yet to be thoroughly studied. In addition, the role of zingerone in cell-cycle arrest and its regulatory mechanisms remain undescribed.

Neuroblastoma most often occurs in babies and children. Although children at low risk are curable after treatment, those at high risk show very low survival rates despite aggressive treatment [15]. Therefore, alternative regimens or supplemental anti-cancer drugs are required for successful treatment of neuroblastoma. In this study, we demonstrated, for the first time, that zingerone significantly suppressed the growth of neuroblastoma cells. Zingerone induced cell-cycle arrest at mitosis concurrently with inhibition of cyclin D1 expression in neuroblastoma cells. Zingerone blocked tumor progression in a mouse tumor model, suggesting that it might be effective for the treatment of neuroblastoma.

## 2. Results

### 2.1. Zingerone Decreases Neuroblastoma Cell Survival

**Figure 1:**
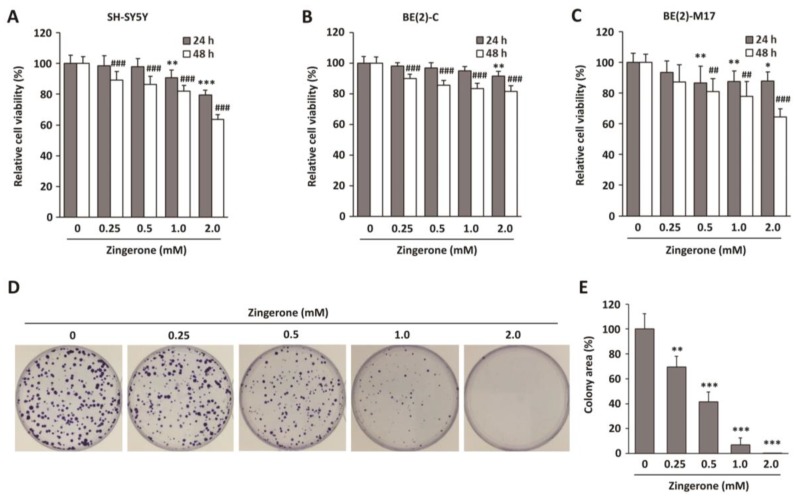
Effects of zingerone on viability of neuroblastoma cell lines. (**A**) SH-SY5Y, (**B**) BE(2)C, and (**C**) BE(2)-M17 cells were treated with zingerone for 24 and 48 h. Survival rate was determined using 3-(4,5-dimethylthiazol-2-yl)-2,5-diphenyltetrazolium bromide (MTT) assays. Data are presented as means ± standard deviation (SD) of the mean. All experiments were done three times independently. * *p* < 0.05; ** *p* < 0.01; and *** *p* < 0.001 vs. vehicle-treated control for 24 h. ^##^ *p* < 0.01 and ^###^ *p* < 0.001 vs. vehicle-treated control for 48 h. (**D**) Colony-formation assays of BE(2)-M17 cells treated with zingerone at the indicated concentration for two weeks. (**E**) Data analysis of colony area of (**D**). The data from three independent analyses are presented as the means ± SD of the mean. * *p* < 0.05; ** *p* < 0.01; and *** *p* < 0.001 vs. vehicle-treated control.

To investigate the possible therapeutic effect of zingerone in neuroblastoma cancer, the effects of zingerone on the viability of human neuroblastoma cells were evaluated. Human neuroblastoma cell lines SH-SY5Y, BE(2)C, and BE(2)-M17 were treated with zingerone for 24 or 48 h, and cell viability was measured using 3-(4,5-dimethylthiazol-2-yl)-2,5-diphenyltetrazolium bromide (MTT) assays. As illustrated in Figure 1A–C, zingerone significantly suppressed cell viability in a dose-dependent manner in all neuroblastoma cell lines we used, whereas it was not cytotoxic for normal cells (Supplementary Materials, Figure S1). Since zingerone exhibited similar effects on these cell lines, we selected BE(2)-M17 cells for further studies. As the long-term effect of zingerone is as important as its short-term effect on tumorigenic activity, we also performed a clonogenic assay. Zingerone-treated BE(2)-M17 cells showed significantly reduced colony-forming activity in a dose-dependent manner (Figure 1D,E). These results suggest that zingerone is a potentially effective therapeutic agent for neuroblostoma cells.

### 2.2. Zingerone Induces Cell-Cycle Arrest at Mitosis

**Figure 2:**
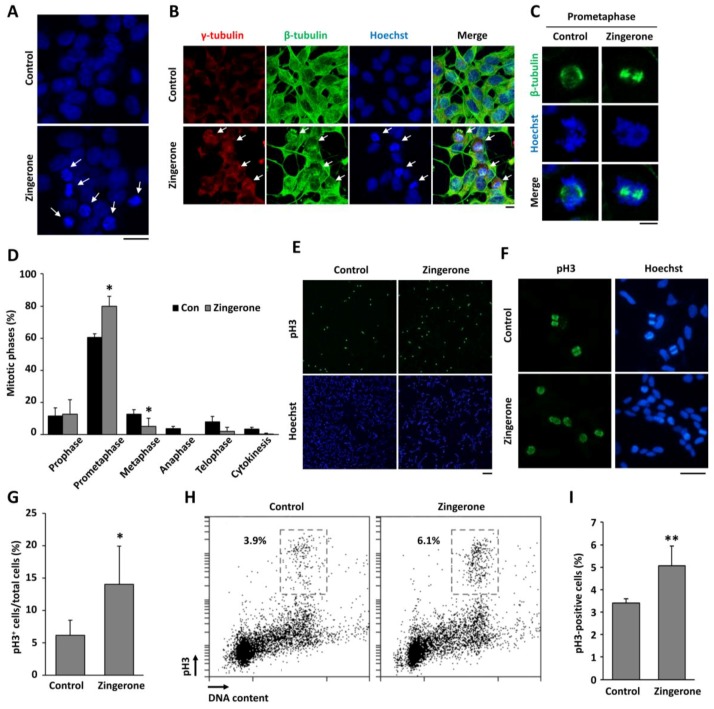
Effects of zingerone on cell-cycle arrest in BE(2)-M17 cells. (**A**) BE(2)-M17 cells were treated with 2 mM zingerone for 24 h. After zingerone treatment, DNA was stained with Hoechst33342 (blue). Arrows indicate condensed chromosomes. The scale bar is 20 μm. (**B**) Cell were treated with 2 mM zingerone for 24 h and then immunofluorescence staining with anti-γ-tubulin (red), β-tubulin (green), and Hoechst33342 (blue) was performed. Arrows indicate mitotic cells. (**C**) In particular, unaligned condensed chromosomes at prometaphase in control (left) and zingerone-treated (right) cells were represented. The scale bar is 5 μm. (**D**) The number of mitotic cells was counted. The data from three independent analyses are presented as the means ± SD of the mean. * *p* < 0.05 vs. vehicle-treated control. (**E**,**F**) After treatment with 2 mM zingerone for 24 h, cells were stained with anti-histone H3 phosphorylated (p)-Ser10 (pH3; green) and Hoechst33342 (blue). (**E**) The cells were observed under a fluorescence microscope. The scale bar is 100 μm. (**F**) Higher magnification of pH3-positive cells. The scale bar is 50 μm. (**G**) The pH3-positive cells were counted and quantified from three independent experiments. (**H**) DNA content and pH3-positive cells were determined by flow cytometry. (**I**) The pH3-positive cells were counted and quantified from three independent experiments. The data are presented as the means ± SD of the mean. * *p* < 0.05; ** *p* < 0.01 vs. vehicle-treated control.

Since many anti-cancer drugs disrupt the progression of the cell cycle [16], we carefully examined the morphology of nuclei in zingerone-treated cells to distinguish between interphase and mitosis. As shown in Figure 2A, the number of cells containing condensed chromosomes was increased by treatment with zingerone for 24 h. Each mitotic cell was distinguished by the location and shape of centrosomes, mitotic spindles, and chromosomes by staining with antibodies for γ-tubulin, β-tubulin, and Hoechst33342, respectively. We found that zingerone induced mitotic arrest (Figure 2B). In particular, cells in prometaphase, which contain unaligned condensed chromosomes, were frequently observed in zingerone-treated cells compared to control cells (Figure 2C). The number of cells in prometaphase, which contain unaligned condensed chromosomes (Figure 2C), was significantly increased, while the number of cells in metaphase, containing aligned chromosomes at the metaphase plate, was significantly decreased by zingerone (Figure 2D). It suggests that zingerone induces a delay in transition from prometaphase to metaphase. By contrast, the number of cells in late mitosis, such as telophase and cytokinesis, was decreased although it was not significant. Overall, it indicates that zingerone arrests cells at prometaphase. To confirm the effect of zingerone on mitotic arrest, we stained cells for histone H3 phosphorylated (p)-Ser10 (pH3), a mitosis-specific marker, and analyzed the number of pH3-positive cells by immunofluorescence cytochemistry (Figure 2F) and cell-cycle analysis (Figure 2H). As expected, the number of pH3-positive cells was significantly increased by zingerone (Figure 2G,I). These findings suggest that zingerone treatment induces mitotic arrest in neuroblostoma cells.

### 2.3. Regulatory Proteins of Mitosis Were Upregulated in Human Neuroblastoma

**Figure 3:**
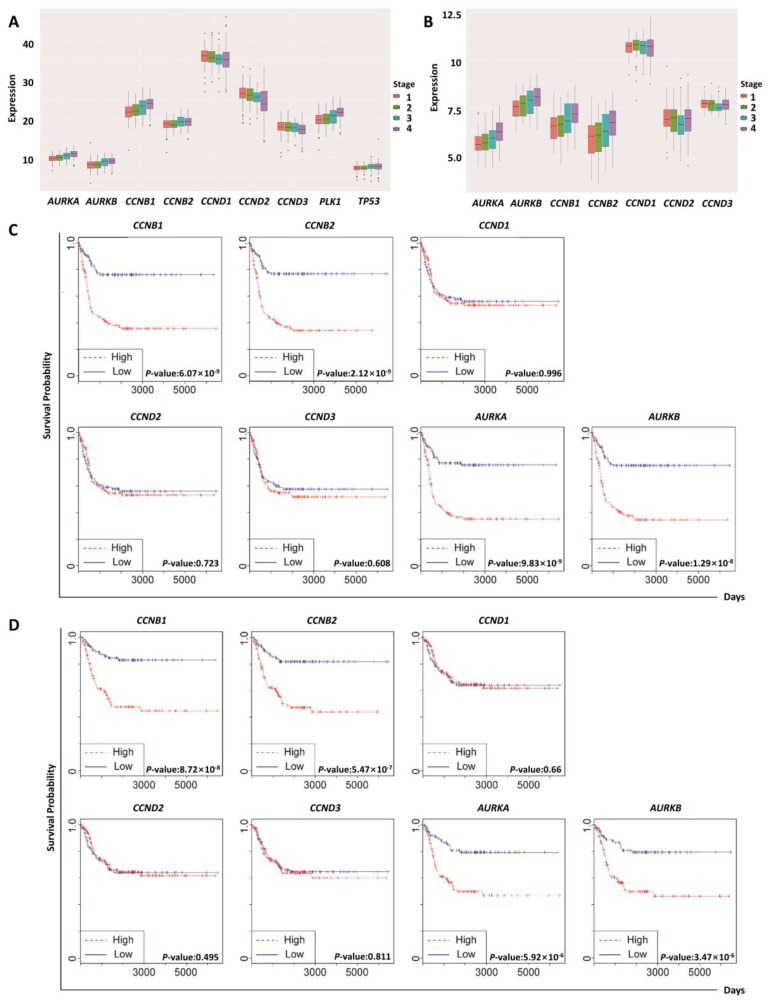
Expression patterns of mitosis-regulatory genes in neuroblastoma patients. (**A**) Gene-expression patterns of *CCNB1*, *CCNB2*, *CCND1*, *CCND2*, *CCND3*, *AURKA*, *AURKB*, *PLK1*, and *TP53* in GSE49710. ANOVA tests for all stages in the genes were performed (*p*-values: *CCNB1*, 2 × 10^−16^; *CCNB2*, 4.57 × 10^−5^; *CCND1*, 0.081; *CCND2*, 3.83 × 10^−9^; *CCND3*, 0.000346; *AURKA*, 2 × 10^−16^; *AURKB*, 1.71 × 10^−13^; *PLK1*, 2 × 10^−16^; *TP53*, 0.000786). The *y*-axis indicates messenger RNA (mRNA) expression. (**B**). Gene-expression patterns of *CCNB1*, *CCNB2*, *AURKA*, and *AURKB* in GSE85047, according to stages (*p*-values of ANOVA tests: *CCNB1*, 3.95 × 10^−7^; *CCNB2*, 7.15 × 10^−6^; *CCND1*, 0.898; *CCND2*, 0.42; *CCND3*, 0.336; *AURKA*, 8.63 × 10^−7^; *AURKB*, 4.62 × 10^−5^. The *y*-axis indicates mRNA expression. (**C**) Progression-free survival plots and log-rank test *p*-values of *CCNB1*, *CCNB2*, *CCND1*, *CCND2*, *CCND3*, *AURKA*, and *AURKB* in GSE85047. (**D**) Overall survival plots and log-rank test *p*-values of *CCNB1*, *CCNB2*, *CCND1*, *CCND2*, *CCND3*, *AURKA*, and *AURKB* in GSE85047.

Cell-cycle proteins are commonly mutated or upregulated in human cancers [1,17,18,19,20,21,22,23]. To identify a mechanistic target of zingerone, we firstly analyzed the correlation between the expression level of mitosis regulators among cell-cycle proteins and clinicopathological features of human neuroblastoma patients, utilizing publicly available datasets. Gene-expression patterns of cell-cycle genes and their association with survival were inspected using the two neuroblastoma Gene Expression Omnibus (GEO) datasets, GSE49710 and GSE85047. *CCNB1, CCNB2,* and *AURKA* expression gradually increased from stages 1 to 4 in GSE49710 (Figure 3A). Moreover, the gene-expression patterns of *CCNB1*, *CCNB2*, *AURKA*, and *AURKB* progressively went up from stages 1 to 4 in GSE85047 (Figure 3B). To investigate whether these cell-cycle regulators are potential targets for cancer therapy, we examined the relationship between expression patterns and the survival rate in neuroblastoma patients performing Kaplan–Meier analysis. As shown in Figure 3C,D, *CCNB1*, *CCNB2*, *AURKA*, and *AURKB* messenger RNA (mRNA) expressions showed significant survival differences between patients with high and low expression of these genes for both datasets. The mRNA expressions of *PLK1* and *TP53* also increased with the stage of neuroblastoma in GSE49710 (Figure 3A). However, we could not assess the survival rates because survival information associated with these genes was unavailable in GSE85047.

### 2.4. Zingerone Reduced Cyclin D1 Expression in Human Neuroblastoma Cells

**Figure 4:**
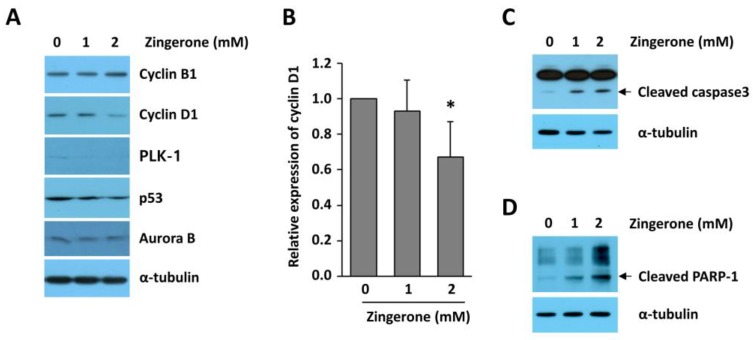
Effect of zingerone on the expressions of cell-cycle-associated proteins and apoptosis in BE(2)-M17 cells. (**A**) BE(2)-M17 cells were treated with the indicated concentration of zingerone for 24 h. Cell lysates were chemiluminescent-immunoblotted with antibodies for cyclin B1, cyclin D1, polo-like kinase 1 (PLK-1), p53, and Aurora B. (**B**) The relative cyclin D1 levels (cyclin D1 expression vs. α-tubulin expression) from three independent experiments were expressed as means ± SD of the mean. The expression in the control was set to 1.0. * *p* < 0.05 vs. vehicle-treated control. (**C**,**D**) BE(2)-M17 cells were treated with the indicated concentration of zingerone for 72 h (**C**) and 96 h (**D**). Cell lysates were immunoblotted with antibodies for caspase-3 (**C**) and poly (ADP-ribose) polymerase 1 (PARP-1; **D**). α-Tubulin was used as a loading control.

Since we already found that the expression of mitosis regulators was increased in human neuroblastoma, we examined which cell-cycle-regulatory genes are regulated by zingerone. As illustrated in Figure 4A,B, zingerone reduced the expression of cyclin D1 in BE(2)-M17 cells. By contrast, the expressions of cyclin B1, Aurora kinase B (Aurora B), polo-like kinase 1 (PLK-1), and p53, all of which are increased from stages 1 to 4 of neuroblastoma patients, were not reduced by zingerone treatment (Figure 4A). Assuming that zingerone induces mitotic arrest, the low expression of cyclin D1 implies that zingerone-treated cells failed to divide into two daughter cells and move to the next cell cycle. To investigate whether zingerone-induced mitotic delay influences apoptotic cell death, we examined cleaved poly (ADP-ribose) polymerase 1 (PARP-1) and cleaved caspase-3. As shown in Figure 4C,D, zingerone strongly increased cleavage of caspase-3 and PARP-1 in BE(2)-M17 cells. Overall, these results demonstrate that zingerone dysregulates cell-cycle progression, and then induces apoptosis in neuroblastoma cells.

### 2.5. Zingerone Inhibits Tumor Progression in a Mouse Tumor Model

**Figure 5:**
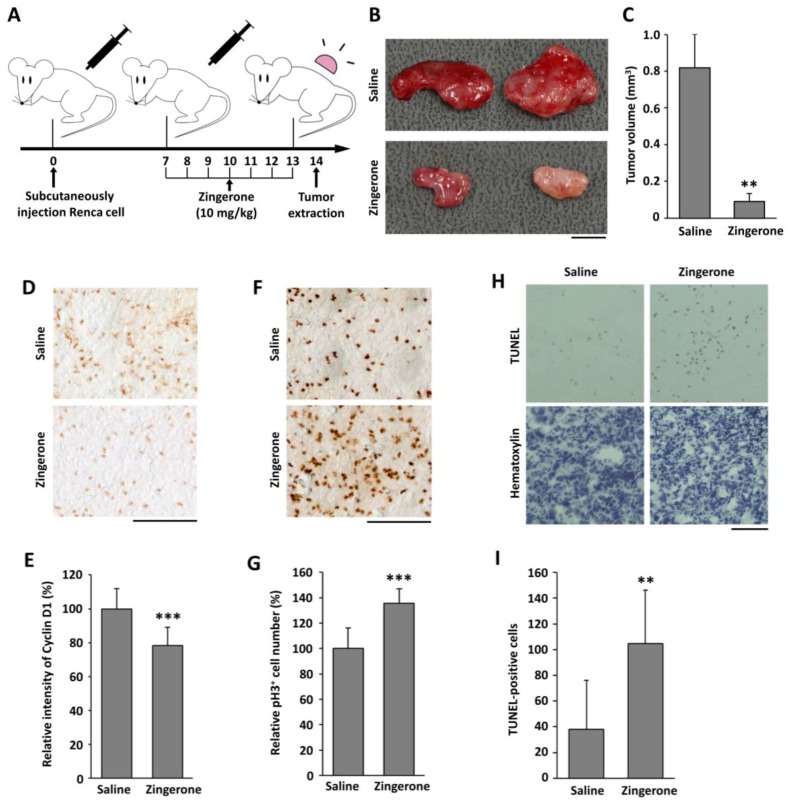
Effect of zingerone on tumor growth and expressions of cell-cycle-regulatory protein in an in vivo mouse tumor model. (**A**) Experimental scheme of the administration. (**B**) Representative picture of tumors from saline-treated mice (upper) and zingerone-treated mice (lower). The scale bar is 0.5 cm. (**C**) Tumor sizes are presented as the means ± SD of three independent experiments with four mice per group. ** *p* < 0.01 vs. saline. (**D**–**G**) Immunostaining for cyclin D1 (**D**) and pH3 (**F**) were performed using isolated tumor tissues. The scale bar is 100 μm. The signals for cyclin D1 (**E**) and pH3 (**G**) were quantified using the ImageJ software (NIH, Bethesda, MD, USA), and the data from three independent experiments are presented as the means ± SD. *** *p* < 0.001 vs. saline. (**H**) Tumor tissues were stained with terminal deoxynucleotidyl transferase deoxyuridine triphosphate (dUTP) nick end labeling (TUNEL; upper) and counterstained with hematoxylin (lower). The scale bar is 100 μm. (**I**) The apoptotic cells were counted and quantified from three independent experiments. ** *p* < 0.05 vs. saline.

To examine the effect of zingerone on tumor growth, we used a BALB/c mouse tumor model, stimulated by the injection of Renca cells originating from a BALB/c strain. Ten days after the injection of the Renca cells, the mice were intraperitoneally administered with zingerone (10 mg/kg) every day for a week (Figure 5A). When the tumors were isolated, the tumor sizes of zingerone-administered mice were significantly smaller than those of the vehicle-treated control group (Figure 5B,C). To investigate whether a decrease in tumor size due to zingerone is associated with a low expression of cyclin D1, we examined the level of cyclin D1 via immunohistochemistry. As shown in Figure 5D,E, the signal for cyclin D1 was reduced in tumors from zingerone-treated mice. Moreover, the number of pH3-positive cells was increased in zingerone-treated tumor tissues (Figure 5F,G). To assess the number of apoptotic cells, a terminal deoxynucleotidyl transferase deoxyuridine triphosphate (dUTP) nick end labeling (TUNEL) assay was performed. The number of TUNEL-positive apoptotic cells in the zingerone-treated group was found to be significantly higher than the control group (Figure 5H,I). This suggests that zingerone blocks tumor progression through mitotic arrest, failure of cell division, and stimulation of apoptosis.

## 3. Discussion

Various therapeutic strategies for human cancers were developed to date. However, the overall efficiency of treatment for malignant and metastatic cancer remains stagnant. Recent studies identified effective phytochemicals for the treatment of cancer [24,25,26,27,28]. We evaluated the potential of zingerone as a therapeutic agent for human cancers, including neuroblastoma. In this study, we found that zingerone, a natural substance, has anti-tumor activities in mice. Tumor development is regulated through the complex actions of tumor cell proliferation and the microenvironment within a developing tumor. In particular, aberrant cell-cycle progression is a common factor triggering uncontrolled proliferation during tumor progression [23,29,30]. Out study demonstrates that zingerone effectively induces mitotic arrest (Figure 2) and suppresses tumor growth in vitro and in vivo (Figure 1 and Figure 5).

Cyclin D1 belongs to the family of D-type cyclins (cyclins D1, D2, and D3) and is expressed in an overlapping, redundant manner in all proliferating cells [31]. Cyclin D1 collectively controls cell-cycle progression by activating the cyclin-dependent kinase partners, CDK4 and CDK6 [31,32,33]. Several mitogenic growth factors increase cyclin D1 levels, and these growth factors are degraded at the end of the S-phase [34]. Overexpression of cyclin D1 was shown to correlate with early cancer onset and tumor progression [35], and can induce chemotherapeutic resistance and protection from apoptosis [36]. The current study shows that zingerone downregulates the expression of cyclin D1 in neuroblastoma cells, implying its action as a potential anti-cancer drug.

Neuroblastoma is a type of cancer that forms in nervous tissue [37]. Commonly, it starts in the adrenal glands, and develops in the neck, chest, abdomen, or spine [37]. This is one of the most common cancers in babies and children [37]. Environmental factors for genetic alterations in neuroblastoma are yet to be found [37]. In this study, we examined expression patterns of genes related to mitosis regulation using neuroblastoma GEO datasets. The gene expression levels of cyclin B1 (*CCNB1*), cyclin B2 (*CCNB2*), Aurora A (*AURKA*), Aurora B (*AURKB*), and PLK-1 (*PLK1*) were increased from stages 1 to 4; however, the genes for D-type cyclins were expressed in a stable manner across the cancer stages (Figure 3). From our results, we could not interpret precisely why cyclin D1 was not upregulated in neuroblastoma. Cyclin D1 is involved in early-onset tumor progression, and *CCND1* represents the second most frequently amplified gene among all human cancer types [38]. We predicted that *CCND1* expression might be maintained at a high rate in all stages, including stage 1 in neuroblastoma. Therefore, we could not detect a gradual incline in *CCND1* expression by staging of neuroblastoma in the datasets GSE49710 and GSE85047.

Recently, natural compounds gained importance as anti-cancer therapeutics due to their nontoxic and anti-carcinogenic activities. Approximately 50% of Food and Drug Administration (FDA)-approved drugs are either natural products, such as phytochemicals, or their derivatives [39]. Our group demonstrated the anti-angiogenic properties of zingerone as a natural compound in tumor models in vitro and in vivo [14]. Zingerone is not only extremely safe in vivo, but also functions through a unique mechanism of action leading to the inhibition of matrix metalloproteinases (MMP)-2 and MMP-9, which are involved in the c-Jun N-terminal kinase (JNK) pathway in tumorigenesis [14]. Deregulated cell-cycle regulators, including mitosis regulators, are implicated in tumor development and poor survival from cancer treatments. Therefore, to determine how zingerone would modulate the cell cycle, we examined the levels of mitotic regulators, cyclins, and mitotic spindle formation. We observed that zingerone inhibited cyclin D1 expression and induced mitotic arrest (Figure 2 and Figure 4). Moreover, the significant reduction in cell proliferation resulting from treatment with zingerone (Figure 1) can also be explained by the induction of apoptosis (Figure 4). Our novel findings may provide new insight into the therapeutic effects of zingerone in cancer treatment through anti-mitotic effects.

## 4. Materials and Methods

### 4.1. Reagents and Antibodies

Zingerone, Hoechst33342, MTT, 3,3-diaminobenzidine, and antibodies for α-tubulin and β-tubulin were purchased from Sigma Aldrich (St. Louis, MO, USA). Hematoxylin solution was purchased from Merck (Darmstadt, Germany). Antibodies for γ-tubulin and pH3 were purchased from NOVUS (Littleton, CO, USA) and Merck (Darmstadt, Germany), respectively. Antibodies against cyclin D1, cyclin B1, PARP-1, PLK-1, Aurora B, p53, and caspase-3 were obtained from Santa Cruz Biotechnology (Santa Cruz, CA, USA). Secondary antibodies conjugated to either Alexa-488 or Alexa-594 were purchased from Invitrogen (Eugene, OR, USA).

### 4.2. Cell Culture and Drug Treatment

Human neuroblastoma BE(2)C, BE(2)-M17, and SY-SY5Y cells and mouse Renca cells were obtained from the American Type Culture Collection (ATCC, Rockville, MD, USA) and maintained in Dulbecco’s modified Eagle’s medium (DMEM; WelGene, Gyeongbuk, Korea) supplemented with 10% fetal bovine serum (FBS; HyClone, Victoria, Australia) and 1% penicillin/streptomycin (Corning Life Sciences, Manassas, VA, USA), in a 5% CO_2_ humidified 37 °C incubator. Cells were treated with 0.25 to 2 mM zingerone for indicated times. Zingerone was dissolved in dimethyl sulfoxide (DMSO) as a vehicle, and the final DMSO concentration for the control was 0.2% (*v*/*v*).

### 4.3. Cell Viability Assay

Initially, 6 × 10^4^ BE(2)-M17, 6 × 10^4^ BE(2)C, and 3 × 10^4^ SH-SY5Y cells were plated per well in 24-well plates and treated with zingerone for 24 and 48 h. MTT was added to each well and incubated at 37 °C for 2 h. After the medium was removed, the formazan crystals were dissolved using DMSO. The absorbance was measured with a test wavelength of 590 nm. All values were expressed as the means ± SD of three wells from three independent experiments.

### 4.4. Colony-Formation Assay

The colony-formation assay was performed as previously described [40]. Briefly, 500 cells were plated in a 60-mm dish and then incubated with 0.25–2 mM zingerone for two weeks. After the medium was removed, cells were fixed with methanol, and then stained using crystal-violet staining solution. Colonies were counted and values were expressed as the means ± SD of three independent experiments.

### 4.5. Immunofluorescent Analysis

Immunofluorescent analysis was performed as previously described [41]. BE(2)-M17 cells were treated with 2 mM zingerone for 24 h and then the cells were fixed with ice-cold 100% methanol for 15 min at −20 °C, before being washed three times with phosphate-buffered saline (PBS) for 5 min. The cells were incubated with blocking solution (5% bovine serum albumin (BSA) and 0.3% Tween-20 in PBS) for 1 h at room temperature and incubated with primary antibodies for pH3, β-tubulin, and γ-tubulin at 4 °C overnight. The cells were incubated with secondary antibodies conjugated to ether Alexa-488 or Alexa-594 for 1 h and then incubated with Hoechst33342 for 10 min at room temperature. Each mitotic phase was distinguished by location and shape of chromosomes, and the mitotic spindles and spindle poles which were stained with Hoechst33342 and anti-β-tubulin antibody. Condensed chromosomes are visible and spindle fibers start growing from the centrosomes at prophase. Chromosomes are more condensed, but not yet fully aligned, and mitotic spindles are attached to the kinetochore at prometaphase. Chromosomes are aligned to the spindle equator and mitotic spindles referred to as K-fibers are tightly attached to individual kinetochores at metaphase. Sister chromatids move toward opposite poles and mitotic spindles are elongated at anaphase. Chromosomes reach spindle poles and begin decondensing, and the mitotic spindle is disassembled at telophase. The nucleus conformation is completely formed and the cytoplasm is separated by furrowing plasmalemma in the equatorial region at cytokinesis [42]. For analysis, 150 cells were randomly selected, and the number of cells with pH3-positivity was counted in a blinded manner. Independent experiments were done three times.

### 4.6. Cell-Cycle Analysis

Cells were fixed with 70% ethanol while gently vortexing. The fixed cells were permeabilized with 0.25% Triton X-100 in PBS on ice for 15 min. The cells were incubated with anti-pH3 (Millipore, Burlington, MA, USA) antibody for 2 h, and then incubated with the corresponding secondary antibody (Jackson ImmunoResearch Laboratories Inc., West Grove, PA, USA) at room temperature in the dark for 1 h. Cells were incubated with DNase-free RNase A at 37 °C for 30 min and then with propidium iodide (PI) at 37 °C in the dark for another 30 min. The percentage of pH3-positive cells was determined by flow cytometry [43].

### 4.7. Gene-Expression Patterns and Survival Analyses of Cell-Cycle Genes

Two publicly available neuroblastoma datasets were obtained from the GEO [44]: GSE49710 [45] and GSE85047 [46]. From the two datasets, GSE49710 and GSE85047, stage 1–4 patients having available survival information were obtained (4S not considered), including 445 and 248 neuroblastoma patients, respectively. The cell-cycle genes of interest were *CCNB1*, *CCNB2*, *CCND1*, *CCND2*, *CCND3*, *AURKA*, *AURKB*, *PLK1*, and *TP53*. For mRNA expression significances of these genes, mRNA expressions were inspected by ANOVA and Tukey’s tests. The dataset GSE85047 included survival information, but the other dataset GSE49710 did not. We performed survival analysis only for GSE85047. In the survival analyses (overall survival and progression-free survival), log-rank tests and Kaplan–Meier plots were performed. In the survival analyses, high- and low-expressing groups of a gene were divided by the median expression of the gene.

### 4.8. Western Blot Analysis

Western blot analysis was performed as previously described [47]. BE(2)-M17 cells were treated with 1–2 mM zingerone for 24 h to 96 h and the cells were harvested in Nonidet P-40 (NP-40) lysis buffer (50 mM Tris-HCl, 150 mM NaCl, 1% NP-40, protease inhibitors, pH 8.0). Extracts were centrifuged at 14,000 rpm for 20 min at 4 °C and then the supernatant was collected. Whole-cell lysate was resolved on a SDS-PAGE gel and transferred to polyvinylidene fluoride (PVDF) membranes. Membranes were incubated with the specific primary antibodies for cyclin D1, cyclin B1, PLK1, Aurora B, PARP-1, caspase-3, p53, and α-tubulin at 4 °C overnight and then with peroxidase-conjugated secondary antibodies. The bands were visualized with the SuperSignal West Pico plus chemiluminescent substrate.

### 4.9. Assessment of Chromosome Condensation

Chromosome condensation was assessed via Hoechst33342 staining. After treatment with zingerone, BE(2)-M17 cells were fixed in ice-cold 100% methanol for 15 min, and washed three times with PBS with Tween-20 (PBST). The cells were incubated with Hoechst33342 for 10 min at room temperature.

### 4.10. Mouse Tumor Model

Male BALB/c mice (six weeks old) were purchased from Daehan Bio-link (Chungbuk, Korea). These experiments were approved by the Committee for Care and Use of Laboratory Animals at the Kyung Hee University (KHUAP(SE)-17-018, 26-04-2017) according to the Guide for Animal Experiments edited by the Korean Academy for Medical Sciences. Renca cells (5 × 10^6^), a BALB/c-derived renal adenocarcinoma cell line, were mixed 1:1 with Matrigel (BD Pharmingen, San Diego, CA, USA) and subcutaneously injected into mice. After seven days, the mice were injected daily intraperitoneally with either zingerone (10 mg/kg in saline) or saline for a week. At 14 days after cell injection, the mice were sacrificed for tumor extraction (Figure 5A). The tumor volume (mm^3^) = 0.5 (width × length × height) [47]. Four mice were used per group, and the experiments were independently repeated three times.

### 4.11. Immunohistochemistry

Tumors extracted from mice were embedded in optimal cutting temperature (OCT) compound, frozen, cut into 30-μm slices using a freezing microtome (Leica Microsystems, Bensheim, Germany), and placed on gelatin-coated slides. The sections were incubated with PBS containing 0.3% Triton X-100 for 30 min, incubated with blocking solution (5% FBS, 5% BSA, and 0.3% Tween-20 in PBS) for 1 h, and stained with specific antibodies against cyclin D and pH3 at 4 °C overnight. The antigen sites were visualized with the VECTASTAIN Elite avidin–biotin complex (ABC) Kit (VECTOR laboratories, Burlingame, CA, USA) and 3,3-diaminobenzidine. The intensity of each staining was measured using the ImageJ software.

### 4.12. TUNEL Assay

For detection of apoptosis, the TUNEL assay was performed using mouse tumor tissues using a TUNEL assay kit (Promega, Madison, WI, USA) according to the manufacturer’s protocol. The nuclei were counterstained with hematoxylin. The TUNEL-stained sections were viewed under a light microscope, and images of nine randomly selected fields were captured at 400× magnification for each slide.

### 4.13. Statistical Analysis

All experiments were performed at least three times. The intensity of cyclin D1 staining from immunohistochemistry, the colony area from the colony-formation assay, and protein expression from Western blot analysis were measured using the ImageJ software (Wayne Rasbang, NIH, Bethesda, MD, USA). The results were expressed as the means ± SD from at least three independent experiments. Statistical analysis was performed using the IBM SPSS software (version 23). Differences between two groups were examined using Student’s *t*-test. Differences between three or more groups were evaluated by one-way analysis of variance (ANOVA) followed by a Tukey’s honest significant difference (HSD). Post hoc tests were run only if F achieved *p* < 0.05 and there was no significant inhomogeneity. Statistical differences were considered significant from the control when *p* < 0.05. 

## Supplementary Materials

The following are available online at http://www.mdpi.com/1422-0067/19/9/2832/s1.

## Abbreviations

CDK	Cyclin-dependent kinase
pH3	Histone H3 phosphorylated Ser10
GEO	Gene Expression Omnibus
PLK-1	Polo-like kinase 1
PARP-1	Poly (ADP-ribose) polymerase 1
FDA	Food and Drug Administration
MMP	Matrix metalloproteinase
JNK	c-Jun N-terminal kinase
FBS	Fetal bovine serum
DMEM	Dulbecco’s modified Eagle’s medium
DMSO	Dimethyl sulfoxide
MTT	3-(4,5-Dimethylthiazol-2-yl)-2,5-diphenyltetrazolium bromide
PBS	Phosphate-buffered saline
PBST	Phosphate-buffered saline with Tween-20
BSA	Bovine serum albumin
OCT	Optimal cutting temperature
ABC	Avidin–biotin complex
ANOVA	Analysis of variance
*CCNB*	Cyclin B
*CCND*	Cyclin D
*AURKA*	Aurora kinase A
*AURKB*	Aurora kinase B
*TP53*	Tumor protein p53
SDS-PAGE	Sodium dodecyl sulfate/polyacrylamide gel electrophoresis
PVDF	Polyvinylidene fluoride
NP-40	Nonidet P-40
TUNEL	Terminal deoxynucleotidyl transferase dUTP nick end labeling
