# Supplementary figures and images for: Zingerone Suppresses Tumor Development through Decreasing Cyclin D1 Expression and Inducing Mitotic Arrest
Source: Int J Mol Sci. 2018 Sep 19;19(9):2832. doi: 10.3390/ijms19092832 (PMC6163242; doi:10.3390/ijms19092832)

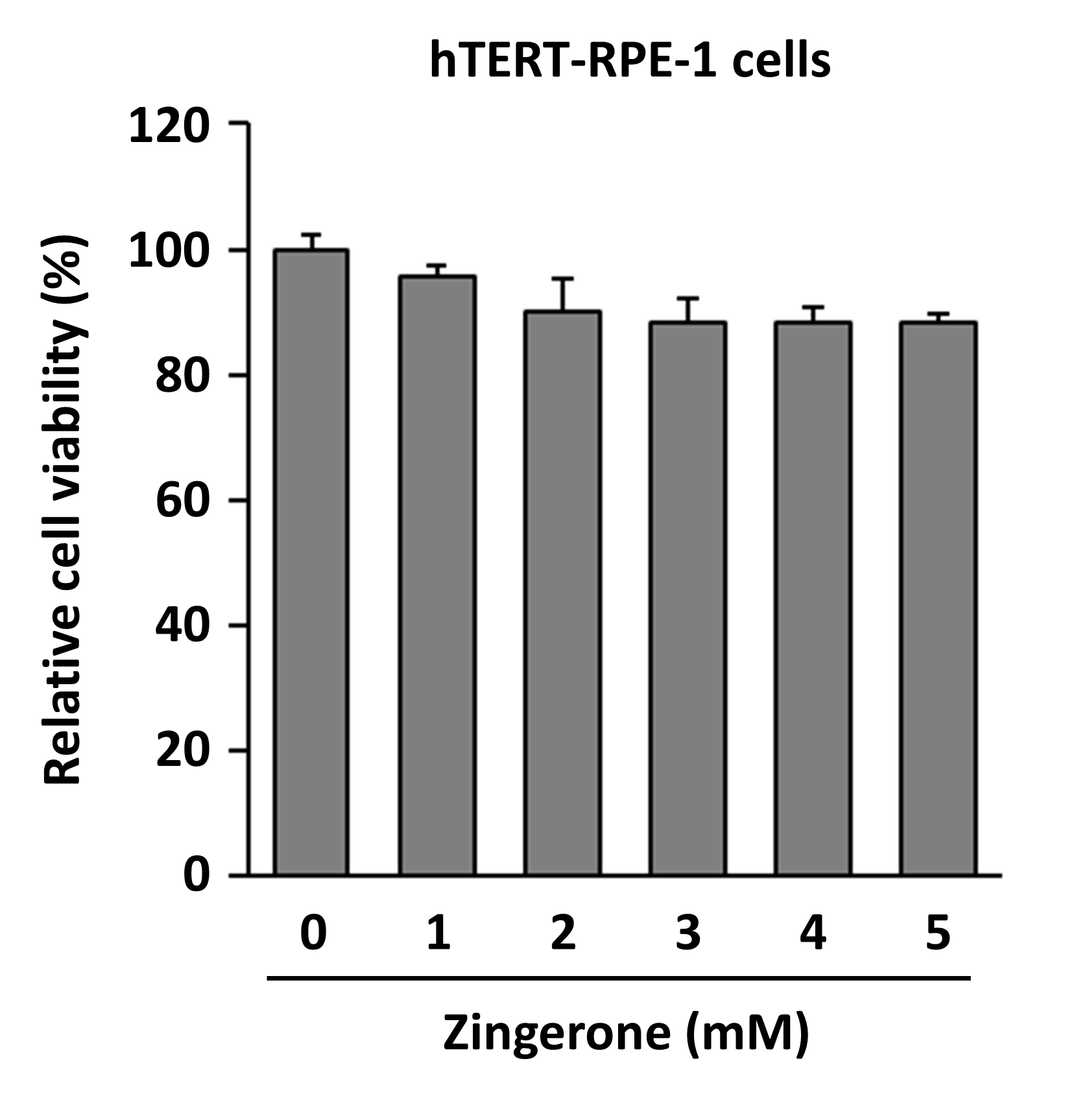

Supplement: Supplementary file 1 [file ijms-19-02832-s001.zip › ijms-343799-SI.tif]
